# Effectiveness of silver diamine fluoride 38% on reduction of gingivitis in dogs: a randomized clinical trial

**DOI:** 10.3389/fvets.2023.1255834

**Published:** 2023-09-20

**Authors:** Amarett Kern, Tammy (White) Renteria, Marilynn L. Rothen, Lloyd A. Mancl, Peter Milgrom

**Affiliations:** ^1^Inland Northwest Veterinary Dentistry and Oral Surgery, Coeur d’Alene, ID, United States; ^2^School of Dentistry and Research Implementation Manager, Institute of Translational Health Sciences Regional Clinical Dental Research Center, University of Washington, Seattle, WA, United States; ^3^Department of Oral Health Sciences, University of Washington, Seattle, WA, United States

**Keywords:** silver diamine fluoride, gingivitis, plaque index, periodontal disease prevention, dog

## Abstract

**Introduction:**

Periodontal disease is a ubiquitous disease in small animal veterinary medicine. Currently regular professional dental cleaning and daily tooth brushing are considered gold standards in the prevention of periodontal disease. Efforts to find a noninvasive, cost effective and easy to use preventative for periodontal disease are ongoing. The primary objective of this double-blind randomized clinical trial was to determine if a single application of silver diamine fluoride (SDF) 38% on the buccal surface of all teeth would reduce gingivitis within 3 months in dogs with stage two periodontal disease.

**Methods:**

Twenty-nine client-owned dogs 3–12 years old, 6–35 pounds were randomized 1:1 into active and placebo-control groups. Both groups underwent a baseline treatment and a three-month follow-up under general anesthesia. Gingival Index (GI), Plaque Index (PI), and Calculus Index (CI) were assessed and recorded at each event.

**Results:**

A single application of SDF 38% did not significantly lower GI relative to the control group. However, the GI score dropped significantly in both groups relative to baseline, with a 53% reduction in the average GI score for dogs that received SDF 38% treatment and a 44% reduction for dogs that received placebo treatment. There were no differences in PI or CI scores compared to control groups.

**Conclusion:**

Further research is needed to determine if a more frequent application or a longer study duration would yield a different outcome.

## Introduction

Periodontal disease is a persistent concern in small animal veterinary medicine. As many as 80 percent of dogs will have gingivitis or periodontitis by 2 years of age (1). Small breed dogs are at higher risk (2) and risk increases with age (3). Periodontal disease is the most common cause of tooth loss in dogs (1) and its potential to be associated with, or the cause of systemic diseases is an ongoing discussion (4–8).

The pathophysiology of early gingivitis and advanced stages of periodontal disease is a result of the complex interplay between bacterial pathogens and the host immune response (5) often exacerbated by crowded dentition or malocclusion. Therapy that targets a rate-limiting step along this continuum could lower the risk of periodontal disease and tooth loss. Development of a therapy that prevents the formation of a pathogenic dental plaque biofilm is potentially one such step. Gingivitis is a reversible inflammatory change and is the first pathological change defined in stage one periodontal disease (9). If plaque accumulation and gingivitis can be reduced, the risk of periodontal disease and tooth loss may be lessened.

Regular professional dental cleanings combined with daily tooth brushing is the gold standard for maintaining oral and dental health (10). Professional dental cleanings pose challenges for many owners because of the cost of therapy and/or fear of general anesthesia. Some pets are refractory to consistent tooth brushing. Owner adherence to brushing their dogs teeth daily has been reported to range from 53% (11) to as low as 1% (12). There are various dental chews or treats available touting oral health benefits. However, these claims are not always supported with valid scientific research. Evidence-based medicine is encouraged in veterinary dentistry to integrate clinically relevant, statistically valid research with clinical expertise and patient needs (13). The Veterinary Oral Health Council (VOHC) provides a central resource for participating veterinary products such as dental chews, treats, food and water additives that align with evidence-based medicine standards. However, even in this setting, the level of evidence of effectiveness may appear low. Common limitations of studies on accepted dental care products are small study populations preventing generalization, short follow-up timelines, and low baseline levels of periodontal disease at enrollment resulting in a ceiling effect (14, 15). Thus, the clinical usefulness of these accepted products remains unclear.

Silver diamine fluoride (SDF) 38% was cleared by the Food and Drug Administration in 2014 as a topical treatment in human dentistry (16). Its use has since become well-accepted to prevent and arrest dental cavities (17) and is included in the World Health Organization (WHO) List of Essential Medicines (18). Its efficacy in preventing and arresting cavities is maintained when applied every 6 to 12 months (19). Additionally, there is new interest with supporting literature regarding its efficacy in preventing and reducing the development of plaque, and subsequently, gingivitis in humans (20, 21).

The potential use of SDF 38% in dogs is extrapolated from its utilization in humans. In dogs there is an overall shift in the oral microbiome from gram-negative bacteria in health to gram-positive bacteria in periodontal disease (22). However, Santibanez et al. also found that *Porphyromonas*, a genus of gram-negative bacteria, is the most abundant species in both healthy and diseased dogs with a 2.7-fold increase in dogs with periodontal disease (23). More specifically, Niemiec et al. found *Porphyromonas gulae* appeared in increasing abundance in the dog’s oral microbiome from health to periodontal disease (22). *P. gulae* is closely related to and shares similar virulence factors to *Porphyromonas gingivalis*, a keystone pathogen in the human oral cavity in periodontal disease (24).

The *Porphyromonas* genus is capable of invading epithelial cells, activating proinflammatory cytokine activity, influencing the innate immune response and exacerbating the progression of periodontal disease (23–25). A human *in-vitro* study by Ho et al. found that SDF 38% was effective at inhibiting the growth of *Porphyromonas gingivalis*, attributed to its bactericidal effects on gram-negative bacteria (26). This supports investigating the use of SDF 38% as a therapy for slowing the progression of periodontal disease in dogs.

Furthermore, when applied to the tooth surface, SDF 38% reacts with hydroxyapatite, producing the metabolites silver phosphate, calcium fluoride and ammonium hydroxide (27). The silver and fluoride metabolites are thought to have a synergistic effect that results in bactericidal activity on gram-positive and gram-negative organisms. Due to the mechanism of action of its active compounds, SDF 38% may show promise in preventing gingivitis in dogs.

While the author is unable to find safety studies conducted specifically in dogs, the pharmacokinetics of SDF 38% have been investigated in human dentistry and found to be safe. Three human pharmacokinetic studies, including one in children, have addressed the potential for small amounts of SDF 38% to be ingested and absorbed through the gastrointestinal tract following application to healthy and diseased teeth. All three studies found there was no significant increase in blood serum levels above pretreatment baseline levels, concluding that SDF 38% application did not pose a toxicity concern (28–30).

The low toxicity risk in human patients following application of SDF 38% to a healthy or diseased tooth surface supported our use of the product in this clinical study. The current study further reduced the potential for ingestion by applying SDF 38% only to the buccal surfaces and by rinsing the oral cavity following one-minute contact time on the tooth surface.

A well-known adverse effect of SDF 38% is its ability to stain the treated site black (26). Mature enamel and non-carious dentin do not stain (19) and staining of gingival tissue does not occur (31). The application to mature enamel in the current study reduced the potential for esthetic concerns associated with staining. An additional clinically relevant adverse effect of SDF 38% is a stinging sensation upon contact with ulcerated or inflamed tissue (19). A repeated finding in the literature is that soft tissue lesions result in minor discomfort and resolve within 48–72 h (26, 30, 32).

SDF 38% has the potential to be a safe and effective preventative therapy or treatment for periodontal disease in veterinary medicine. The objective of this study was to evaluate the effectiveness of SDF 38% treatment in periodontal disease susceptible dogs to reduce gingivitis and plaque. The hypothesis tested was that professional dental cleaning plus topical treatment with SDF 38% would be more effective in reducing gingivitis and plaque than professional dental cleaning alone. The study was a double-blind randomized, placebo-controlled trial.

## Materials and methods

### Animals

Client-owned dogs were selected by the same two operators (a Diplomate of the American Veterinary Dental College and a part-time resident of the American Veterinary Dental College). Owners provided written informed consent. The study was reviewed and approved by the University of Washington Animal Care and Use Committee (Protocol 4,526–01, 6/24/21) after consideration of the risks and benefits.

### Setting

Enrollment and treatment were carried out at two facilities: Location site one: A dentistry/oral surgery referral practice, Inland Northwest Veterinary Dentistry and Oral Surgery, Coeur D’Alene, Idaho. Location site two: A general practice clinic, Lower Columbia Veterinary Clinic in Longview, Washington. Recruitment began on September 14, 2021, and the last follow-up data were collected on November 01, 2022.

### Inclusion/exclusion criteria

Enrollment in the study was based on visual oral examinations done on awake dogs at the recruitment examination. The included dogs were required to have no worse than stage two periodontal disease, as defined by the American Veterinary Dental College (33) (Table 1) and a modified Löe and Silness Gingival Index around two (34). Recruited dogs were required to have most of their teeth, including; right maxillary third incisor, right maxillary canine, right maxillary third premolar, right maxillary fourth premolar, right maxillary first molar, left maxillary third incisor, left maxillary canine, left maxillary third premolar, left maxillary fourth premolar, left maxillary first molar, right mandibular canine, right mandibular third premolar, right mandibular fourth premolar, right mandibular first molar, left mandibular canine, left mandibular third premolar, left mandibular fourth premolar and left mandibular first molar (35).

**Table 1:** Stages of periodontal disease used in this study.^a^

PD0	Normal	Gingival inflammation of periodontitis is not clinically evident.
PD1	Gingivitis	Gingivitis only without attachment loss; the height and architecture of the alveolar margin are normal.
PD2	Early periodontitis	Less than 25% of attachment loss or, at most there is a stage one furcation involvement in multirooted teeth. There are early radiographic signs of periodontitis. The loss of periodontal attachment is less than 25% as measured either by probing of the clinical attachment level, or radiographic determination of the distance of the alveolar margin from the cementoenamel junction relative to the length of the root.
PD3	Moderate periodontitis	25–50% of attachment loss as measured either by probing of the clinical attachment level, radiographic determination of the distance of the alveolar margin from the cementoenamel junction relative to the length of the root, or there is a stage two furcation involvement in multirooted teeth.
PD4	Advanced periodontitis	More than 50% of attachment loss as measured either by probing of the clinical attachment level, or radiographic determination of the distance of the alveolar margin from the cementoenamel junction relative to the length of the root, or there is a stage three furcation involvement in multirooted teeth.

^a^As defined by the American Veterinary Dental College (33).

To be included the dog needed to have an American Society of Anesthesia score no greater than two (36). A minimum database consisting of a general physical examination (heart and lung auscultation, abdominal palpation, body temperature and weight recorded), evaluation of a complete blood count and chemistry panel and an oral health assessment was completed to assess each dog’s overall health prior to acceptance into the study.

A dog was excluded if systemic immunosuppressants, antibiotics or localized periodontal medications, including Doxirobe gel® (doxycycline hyclate 8.5%) had been administered within the past 3 months of beginning the study. Additional exclusion criteria included receiving over the counter 1-TetraDecanol Complex supplements, other VOHC approved supplements, treats, additives, or oral rinses that might impact the condition of the periodontium within the past 3 months. Dogs that received regular teeth brushing or those that were fed prescription dental diets within the past 3 months were also excluded. No dental therapies, other than those described in the study were permitted during the duration of the dog’s participation in the study.

### Animal review parameters and physical examination schedule

Prior to each session, a general physical examination including heart and lung auscultation, abdominal palpation, body temperature, oral health and body weight was performed and recorded. A brief history was collected from the owner and recorded to confirm each dog was clinically healthy and the owner had no new concerns following their recruitment visit/examination. Physical examination parameters and history were collected and recorded by the same operator (AK).

### Anesthesia protocol

The anesthesia protocol and patient monitoring were performed identically at each facility, as well as at each therapy session. Each dog received gabapentin 50 mg/mL; 5–7.5 mg/kg by mouth upon admission to the clinic the morning of their procedure to reduce anxiety. Premedication was a selection/combination of; Methadone 10 mg/mL: 0.2–0.4 mg/kg intramuscularly, or Butorphanol 10 mg/mL: 0.2–0.4 mg/kg intramuscularly and Dexmedetomidine 500mcg/ml: 2-4mcg/kg intramuscularly. Following appropriate time to onset of action for the selected premedication, a 20- or 22-gauge intravenous catheter was placed in the right or left cephalic vein. Maropitant 10 mg/mL: 1 mg/kg was given slowly over 5 min intravenously. The patient was preoxygenated with flow-by oxygen for 5 min prior to induction with Propofol 10 mg/mL: 4 mg/kg intravenously to effect. After induction, endotracheal intubation was performed, and general anesthesia was maintained with 2% Sevoflurane. Local anesthesia was utilized when teeth were extracted or when periodontal therapies were provided. Bupivacaine (5 mg/mL): 1-2 mg/kg total volume. Each patient’s vitals were monitored and recorded during general anesthesia. Parameters included electrocardiogram, respiratory rate, oxygen percent saturation, temperature, end tidal CO_2_ and systolic/diastolic and mean arterial pressure. A Doppler was used to monitor systolic blood pressure in dogs under 10 kg for improved accuracy. Thermoregulation support was provided. Few variations of this anesthesia protocol were employed, and no emergency drugs were needed. All parameters and remarks were recorded on an anesthesia chart at each treatment session.

### Measures

Modified Löe and Silness Gingival Index (GI), modified Logan and Boyce Plaque Index (PI) and the Warrick and Gorrel Calculus Index (CI) were collected (34) (Table 2). Disclosing solution (HurriView II®, Beutlich Pharmaceuticals, LLC, Bunnell, FL) was used to record the PI by applying it to the buccal surfaces of study teeth and gently rinsing after 10 s. Parameters were recorded for the buccal surface only of the following teeth; right maxillary third incisor, right maxillary canine, right maxillary third premolar, right maxillary fourth premolar, right maxillary first molar, left maxillary third incisor, left maxillary canine, left maxillary third premolar, left maxillary fourth premolar, left maxillary first molar, right mandibular canine, right mandibular third premolar, right mandibular fourth premolar, right mandibular first molar, left mandibular canine, left mandibular third premolar, left mandibular fourth premolar and left mandibular first molar (35). If the dog was missing a target tooth, it was replaced by a tooth of similar function caudal or rostral to it when possible. The baseline and three-month follow-up GI, PI, and CI were assessed by the same experienced examiner (MLR), who completed the examination assisted by the same experienced recorder (MKH). At follow-up, neither the examiner nor the recorder had access to the pretreatment scores. A complete oral examination and dental therapies by the attending veterinarian were delayed until after research parameters were collected and recorded. Intraoral dental radiographs were taken prior to recording the research parameters to determine stage of periodontal disease and whether a study tooth would require extraction during treatment.

**Table 2:** Scoring systems used in this study.

Modified Löe and Silness Gingival Index^a^	Code	Gingival inflammation	Criteria for gingival inflammation
	0		None observed
	1	Slight	Slight redness but no bleeding on probing
	2	Mild	Slight redness and swelling, with delayed bleeding on probing
	3	Moderate	Red, swollen, and bleeds on probing
	4	Severe	Red, swollen, spontaneous or profuse hemorrhage on probing and/or ulceration

Modified Logan and Boyce Plaque Index^b^	Code	Coverage score %	Thickness score (color)
	0	None observed	None observed
	1	<25	Light: Pink to light red
	2	25–49	Medium: Red
	3	50–75	Heavy: Dark red
	4	>75	

Warrick and Gorrel Calculus Index^c^	Code	Coverage score %	Thickness score (mm)
	0	None observed	None observed
	1	<25	<0.5
	2	25–49	0.5–1
	3	50–75	>1
	4	>75	

^a^Gingival index; buccal surface of each is tooth divided in thirds (mesial, buccal, distal). The average of the three sites is the tooth score. The mean of all tooth scores is the gingival index score for a dog. ^b^Plaque and ^c^Calculus indices: the buccal crown surface is horizontally divided; the gingival and coronal segments are each scored for coverage and thickness. These are multiplied for each segment. Scores for the gingival and crown segments are then added for the tooth score. The plaque or calculus index score for a dog is the mean of all tooth scores for a given index.

### Randomization and allocation

Separate randomization lists were generated for each site by the study biostatistician. Dogs were randomized 1:1 to treatment arms.

### Blinding

The owners, study personnel, including treating clinician, examiner and recorder, and the biostatistician were blind to treatment allocation.

### Test products

The active treatment was 38% aqueous silver diamine fluoride solution (fluoride 5%, silver 25%, Advantage Arrest^R^, Elevate Oral Care LLC, West Palm Beach, FL). The comparator treatment was identical except that it contained neither fluoride nor silver. Test and placebo solutions were prepared by Cascade Chemistry, Eugene, OR and a certificate of analysis was obtained from the laboratory certifying solution contents.

### Masking

The test drugs were packaged in identical bottles labeled A or B and drug codes were retained by the laboratory until data analysis had been completed.

### Application protocol

Solutions A or B were applied in an identical manner by the same operator following appropriate training (AK). The amount of Solutions A and B applied followed manufacturer recommendations. The buccal surface of each tooth was gently dried using the dental unit air/water syringe. To apply the solution, a manufacturer supplied microbrush was dipped into the solution and applied to the buccal surface of the crown of every tooth, with focus on the free gingival margin. The solution was left on the tooth surface for 1 min and then the surface was rinsed with distilled water for 1 min.

### Baseline treatment

Following data collection, a comprehensive oral examination including periodontal probing, notation of oral pathology, and review of intraoral dental radiographs taken prior to data collection was completed. Then, necessary therapies, limited to extractions, closed root planing and post-operative intraoral dental radiographs (if indicated) were performed by the same operator (AK). A professional dental cleaning including ultrasonic scaling, hand scaling of each tooth, followed by polish and a final oral rinse with dental unit air/water syringe was completed. Following completion of oral therapies, either Solution A or B was applied topically to the buccal surface of all teeth.

Owners were instructed to not perform at home dental therapies, including tooth brushing until after the three-month follow-up. If extractions were done, owners were instructed to monitor for complications. A recheck examination was advised if signs of inflammation, infection, oral malodor, or discomfort developed.

### Three-month follow-up

Three months, mean (SD) = 3.2 months (0.4), following the initial treatment each dog returned for the second session. Study parameters were collected under general anesthesia in the same manner as at baseline. Following data collection a professional dental cleaning including ultrasonic scaling, hand scaling of each tooth, followed by polish and a final oral rinse with dental unit air/water syringe was completed.

If extractions were done at baseline treatment, confirmation of oral surgery healing was completed at the three-month follow-up. The owner was encouraged to initiate at-home dental care, with daily tooth brushing being the primary recommendation.

## Statistical analysis

### Sample size calculation

The primary outcome measure was the GI score at 3 months post-treatment. A recent study by Mateo et al. (34) showed a 20% reduction in gingivitis scores, 15% reduction in plaque scores and 35% reduction in calculus scores from a dental chew as compared to no intervention. The Mateo study and a similar study (15) enrolled dogs with very low levels of gingivitis creating a ceiling on potential effectiveness. In this study we enrolled dogs with established gingivitis, and hence, expected larger reductions. With a sample size of 15 dogs per intervention, power was 80% to demonstrate a 27% reduction in gingivitis, 25% reduction in plaque and 67% reduction in calculus due to the SDF 38% intervention as compared to control intervention, based on a two-sided two-sample t-test using a 0.05 significance level.

### Analysis

The GI, PI and CI scores for each dog were calculated as the mean score of all scored teeth. Descriptive statistics, including mean, standard deviation, minimum and maximum values, median and 25th and 75th percentiles, are reported by intervention for gingivitis, plaque, and calculus scores at baseline, and 3 months post-treatment. The distribution of the outcomes was assessed by the skewness, normal quantile-quantile plots, and the mean and median. The distributions were symmetrical, and outcomes were approximately normally distributed.

Age and weight of the dogs at baseline were compared between treatments using Welch’s t-test and the number of teeth was compared using Fisher’s exact test. The outcomes were compared between treatments at baseline and 3 months using Welch’s t-test and within each treatment the change between baseline and 3 months was compared using paired t-tests. Linear regression with heteroskedasticity robust standard errors was used to compare outcomes at 3 months adjusting for baseline values of the outcomes, age, and weight and the follow-up time between baseline and 3 months (37). Additional analyzes evaluated the impact of potential outliers given the small sample size.

Similar analyzes were performed for plaque and calculus scores. A two-sided 0.05 significance level was used to determine statistical significance and 95% confidence intervals (C.I.) were reported for all treatment effects to describe the precision of the treatment effect estimates. All statistical analyzes were performed using R statistical software (Version 4.2.0). R Core Team (2022). R: A language and environment for statistical computing. R Foundation for Statistical Computing, Vienna, Austria. URL https://www.R-project.org/.

## Results

Thirty dogs, 3 to 12 years old, female (*n* = 20), male (*n* = 10), weighing 6.25 to 34.6 pounds were enrolled. One dog was eliminated at the end of the study due to advanced periodontal disease found under general anesthesia, leaving 29 dogs in the final analysis. The number of teeth each dog had was similar between both treatment groups. Treatment solution A (*n* = 15) had five dogs with one or more teeth extracted (four dogs with one tooth extracted and one dog with three teeth extracted). Treatment solution B (*n* = 14) had five dogs with one or more teeth extracted (three dogs with one tooth extracted and two dogs with two teeth extracted). When groups were combined, the number of study teeth present ranged from 18/18 teeth in 83% of dogs enrolled (24/29) to 14/18 teeth in 3.4% of dogs enrolled (1/29) (Table 3). Fifteen breeds were represented: terrier/mix (*n* = 5), chihuahua/mix (*n* = 6), other (*n* = 18) including 19 females (18 spayed) and 10 males (7 castrated). Neither age, weight, sex, or breed was associated with GI, PI, or CI.

**Table 3:** SDF 38% treated, and placebo treated dogs: age, weight, and number of teeth.

Characteristic	Total (*N* = 29)	SDF 38% solution treatment group (*N* = 15)	Placebo solution treatment group (*N* = 14)	*p*-value
Age, y				0.88^1^
Mean (SD)	7.1 (2.6)	7.1 (3.1)	7.1 (2.2)	
Range	3 to 12	3 to 12	4 to 11	
Weight				0.40^1^
Mean (SD)	18.0 (6.9)	19.1 (7.0)	16.8 (6.9)	
Range	6.2 to 34.6	8.9 to 34.6	6.2 to 30.1	
Number of teeth, *n* (%)				0.46^2^
14	1 (3.4%)	1 (6.7%)	0 (0%)	
17	4 (13%)	1 (6.7%)	3 (21%)	
18	24 (80%)	13 (87%)	11 (79%)	

^1^Welch’s *t*-test. ^2^Fisher’s Exact Test.

### Site location and treatment allocation

Nine dogs were treated at Inland Northwest Veterinary Dentistry and Oral Surgery: 33% (5/15) of dogs were allocated to the SDF 38% treatment group and 29% (4/14) of dogs were allocated to the placebo treatment group at this location. Twenty dogs were recruited and treated at Lower Columbia Veterinary Clinic, 67% (10/15) of dogs were allocated to the SDF 38% treatment group and 71% (10/14) of dogs were allocated to the placebo treatment group at this location.

### Gingival index – primary outcome

The GI was similar between both groups of dogs at baseline following treatment group allocation and prior to application of either SDF 38% (Solution A) or placebo (Solution B). At baseline the GI for dogs who received SDF 38% was, mean (SD) = 1.7 (0.5), versus GI for dogs who received placebo treatment, mean (SD) = 1.8 (0.4; *p* value = 0.82).

The GI remained similar between SDF 38% treated dogs and placebo treated dogs at the three-month follow-up examination. GI for dogs who received SDF 38% treatment, mean (SD) = 0.8 (0.4), and GI for dogs who received placebo treatment, mean (SD) = 1.0 (0.4; *p* value = 0.32). There was no difference between treatment groups when adjusted for baseline GI, age, weight, sex or breed and follow-up time [difference (SDF – placebo) = −0.05, 95% C.I., −0.3 to 0.2, *p* value = 0.72].

However, there was a significant reduction in GI for both the SDF 38% treated dogs and the placebo treated dogs at the three-month follow-up examination (*p* values <0.0001) with a 53% reduction in the average GI score for dogs that received SDF 38% treatment and a 44% reduction for dogs that received placebo treatment (Table 4).

**Table 4:** Primary outcome: comparison of Gingival Index at baseline and three-month follow-up between SDF 38% and placebo treated dogs.

Gingival Index Modified Löe and Silness	SDF 38% solution treatment group (*N* = 15)	Placebo solution treatment group (*N* = 14)	*P* value^1^
Gingivitis at baseline			
Mean (SD)	1.7 (0.5)	1.8 (0.4)	0.82
Median (IQR)	1.6 (1.4, 2.0)	1.7 (1.5, 2.0)	
Range	1.1 to 2.6	1.0 to 2.7	
Gingivitis at 3-month follow up			
Mean (SD)	0.8 (0.4)	1 (0.4)	0.32
Median (IQR)	0.9 (0.5, 1.0)	1.0 (0.7, 1.1)	
Range	0.2 to 1.7	0.3 to 1.9	
*P* value^2^	<0.0001	<0.0001	
% Change	−53%	−44%	

^1^Welch’s *t*-test *p* value comparing SDF 38% solution treatment group to placebo solution treatment group. ^2^Paired *t*-test *p* value comparing baseline and 3 months within each treatment group. Adjusted for age, weight, sex and breed.

### Plaque index – secondary outcome

The PI was also similar between both groups of dogs at baseline following treatment group allocation and prior to application of either SDF 38% or placebo solution. Dogs that received SDF 38% treatment had a PI of mean (SD) = 8.9 (2.0), and dogs that received placebo treatment had a PI of mean (SD) = 9.2 (2.4; *p* value = 0.71).

At the three-month follow-up examination the PI was lower in placebo treated dogs, mean (SD) = 7.7 (1.8), than in SDF 38% treated dogs, mean (SD) = 9.2 (2.2; *p* value = 0.055) but the difference was no longer significant when adjusted for baseline PI, age, weight, and follow-up time [difference (SDF – placebo) = 1.4, 95% C.I., −0.2 to 3.0, *p* value = 0.093].

At the three-month follow-up examination there was non-significant reduction of 3% for SDF 38% treated (*p* value = 0.065) dogs and non-significant reduction of 16% for the placebo condition (*p* value = 0.079) (Table 5).

**Table 5:** Secondary outcomes: comparison of plaque and calculus scores at baseline and three-month follow-up between SDF 38% and placebo treated dogs.

Characteristic	SDF 38% solution treatment group (*N* = 15)	Placebo solution treatment group (*N* = 14)	*P* value^1^
Plaque at baseline			
Mean (SD)	8.9 (2.0)	9.2 (2.4)	0.71
Median (IQR)	8.8 (7.7, 10.1)	9.2 (7.3, 10.0)	
Range	4.9 to 12.7	5.7 to 13.9	
Plaque at 3-month follow up			
Mean (SD)	9.2 (2.2)	7.7 (1.8)	0.055
Median (IQR)	9.5 (7.8, 10.6)	7.0 (6.7, 8.4)	
Range	4.9 to 14.3	4.9 to 11.4	
*P* value^2^	0.65	0.079	
% Change	3%	−16%	
Calculus at baseline			
Mean (SD)	5.9 (2.5)	7.4 (2.9)	0.16
Median (IQR)	4.9 (4.5, 6.6)	8.8 (4.8, 9.1)	
Range	2.5 to 12.1	1.9 to 11.8	
Calculus at 3-month follow up			
Mean (SD)	3.2 (1.6)	2.5 (1.3)	0.21
Median (IQR)	3.1 (1.6, 4.1)	2.4 (1.8, 3.0)	
Range	1.4 to 6.0	0.6 to 4.7	
*P* value^2^	0.0020	<0.0001	
% Change	−46%	−66%	

^1^Welch’s *t*-test *p* value comparing SDF 38% solution treatment group to placebo solution treatment group. ^2^Paired *t*-test *p* value comparing baseline and 3 months within each treatment group. Adjusted for age, weight, sex, and breed.

### Calculus index – secondary outcome

At the baseline examination following treatment group allocation and prior to application of either SDF 38% solution or placebo solution, there was an insignificant difference in the CI for dogs that received SDF 38% solution treatment, mean (SD) = 5.9 (2.5), compared to dogs that received placebo solution treatment, mean (SD) = 7.4 (2.9; *p* value = 0.16).

At the three-month follow-up examination, the CI was lower in placebo solution treated dogs, mean (SD) = 2.5 (1.3), than in the SDF 38% solution treated dogs, mean (SD) = 3.2 (1.6), but the difference was not statistically significant (*p* value = 0.21). When adjusted for baseline CI, age, weight and follow-up time, the CI was also not significantly lower in the placebo solution treated dogs than in the SDF 38% treated dogs [difference (placebo – SDF) = 1.1; 95% C.I., −0.4 to 2.5; *p* value = 0.14].

There was a significant reduction in CI for both SDF 38% solution treated dogs (*p* value = 0.0020) and placebo solution treated dogs (*p* value = <0.0001), with a 46% and 66% reduction in the average CI score for each, respectively (Table 5).

### Harms

Specific endpoints for harms including clinical signs indicative of discomfort from gingival irritation, any oral surgery complications and tooth staining were evaluated at a follow-up phone call within 24 h following the baseline treatment and the three-month follow-up itself. Follow-up phone calls were conducted by veterinary support staff with instructions to report concerns to the attending veterinarian (AK).

No owners reported clinical signs indicative of oral discomfort or concerns with staining of teeth at the follow-up call the day after the baseline treatment. Oral surgery healing was confirmed at the three-month follow-up. Owners were instructed to reach out with concerns prior to this visit as needed. No oral surgery complications were reported in the current study population.

Overall, no harms were reported, and no animals were withdrawn by the owner or exited by the attending veterinarians for medical reasons or harms associated with the study drug.

## Discussion

The ability of SDF 38% to reduce gingivitis in humans is a growing area of interest (20, 21). Its potential use in dogs to reduce gingivitis is supported by parallels in the microbiome of the human oral cavity and the dog oral cavity at points along the spectrum of health to periodontal disease (23–25), as well as the ability of SDF 38% to mitigate these organisms’ effects without causing harm to the patient (28, 30, 31). Unfortunately, this study was not able to confirm SDF 38% was an effective treatment in dogs for the prevention of gingivitis when SDF 38% treatment was applied after a professional dental cleaning compared to a professional dental cleaning alone.

A possible explanation for our failure to find a primary treatment effect may lie in the current study’s design. The protocols utilized in the papers looking at efficacy in reducing gingivitis in humans employed more frequent application, up to once weekly for multiple weeks (21). This study utilized a protocol with a single application. It is possible a study designed with more frequent applications would yield a higher reduction in gingivitis.

Silver ions have both antimicrobial and bactericidal capacity. The silver ions remain unchanged after application in dead bacterial cells which act as a reservoir for the silver particles. When these ions are released, they are bactericidal to organisms in proximity (38). Despite the ability of SDF 38% to penetrate enamel to a depth of 25 microns (32), its ability to implement its antimicrobial and bactericidal effects on a dog’s healthy tooth are not yet clear. It may be that this product is best applied to teeth that have not recently had a professional dental cleaning, as was demonstrated in the study by Alshehri et al. (21).

It is unclear whether the modified Gingival Index for humans is reliable and valid in dogs. One such variable impacting reliability and validity of measurement is gingival pigmentation. A human dentistry study by Eid et al. found that the degree of melanin induced pigmentation in the gingiva has a negative correlation with the degree of bleeding on probing (39). However, there are no known studies evaluating whether pigmentation influences bleeding on probing in dogs. Additionally, the thinner gingiva in humans has been found to bleed more readily (40). Similarly, small breed dogs have thinner gingiva compared to breeds with higher body weights and this has been correlated with increased risk of periodontal disease in small breeds (41). The author is unaware of literature that compares the thickness of dog gingiva to human gingiva. A comparative pathology study focused on evaluating the differences in human and dog gingiva could elucidate whether the human derived Gingival Index is appropriate for use in dog studies.

The results of the current study suggest that regular professional dental cleanings are an effective therapy in reducing gingivitis. This is in accordance with the current standard of care for veterinary medicine when managing periodontal disease (14, 42).

Additional areas of interest include whether SDF 38% could be a beneficial treatment for enamel hypomineralization in the dog. Enamel hypomineralization increases the risk of dentin sensitivity, periodontal disease, pulpitis, pulp necrosis, and dental trauma (43). In human dentistry SDF 38% has been shown to prevent enamel lesions from extending into the dentin (19). It would be interesting to evaluate if this benefit can be replicated in cases of enamel hypomineralization in the dog.

### Limitations

In the current study dogs were recruited based on awake visual oral examinations. The limitations of diagnosing the stage of periodontal disease accurately with an awake visual oral examination are known. Wallis et al. highlighted this by reporting a disparity between awake visual oral examinations versus oral examinations under anesthesia and the prevalence of periodontitis. The prevalence of periodontitis based on an awake visual oral examination reported levels of 9.3–18.2% whereas a higher prevalence of periodontitis ranging from 44 to 100% was found when the dogs were examined under anesthesia or post-mortem (4). Bauer et al. found that agreement between awake visual oral examinations and oral examinations under general anesthesia, including periodontal probing and intraoral dental radiographs, was weakest for stages zero through two of periodontal disease (44). The awake visual oral examination provided a challenge to this study in adhering to our baseline inclusion criteria of limiting recruitment to stage two periodontal disease. Once under general anesthesia three recruited dogs were found to require extraction of more than one tooth (two dogs – two teeth; one dog – three teeth).

Rejecting these patients from the study following general anesthesia was agreed to be unreasonable as they still had a sufficient number of target teeth for evaluation. For dogs that had teeth extracted, the two-week recheck examination following oral surgery to evaluate for any postoperative complications was not done, potentially underestimating the rate of dehiscence or other side effects.

The three-month duration of the current study may be a limiting factor. A shorter duration was considered and eliminated due to resistance from owners to closely repeat anesthetic events of their pets. A longer duration was not chosen because of potential loss to follow-up. A study design with more frequent rechecks and a longer duration may have yielded different outcomes.

## Data availability statement

The original contributions presented in the study are included in the article/supplementary material, further inquiries can be directed to the corresponding author.

## Ethics statement

The animal studies were approved by University of Washington Animal Care and Use Committee (Protocol 4526–01, 6/24/21). The studies were conducted in accordance with the local legislation and institutional requirements. Written informed consent was obtained from the owners for the participation of their animals in this study.

## Author contributions

AK: Investigation, Resources, Writing – original draft, Writing – review & editing. TR: Project administration, Resources, Writing – review & editing. MLR: Data curation, Investigation, Resources, Supervision, Writing – review & editing. LM: Data curation, Formal analysis, Resources, Software, Validation, Visualization, Writing – review & editing. PM: Conceptualization, Funding acquisition, Project administration, Supervision, Visualization, Writing – review & editing.

## Funding

The study was sponsored by Elevate Oral Care LLC., West Palm Beach, FL. through an unrestricted donation to the University of Washington and direct payment of travel expenses for the investigators to the two sites. Elevate Oral Care provided the test and placebo solutions at no-cost to the study. No Elevate Oral Care personnel had any contact with the participants or had any role in the analysis or interpretation of the study data.

## Conflict of interest

PM is a member of Advantage Silver Dental Arrest, LLC.

The remaining authors declare that the research was conducted in the absence of any commercial or financial relationships that could be construed as a potential conflict of interest.

## Publisher’s note

All claims expressed in this article are solely those of the authors and do not necessarily represent those of their affiliated organizations, or those of the publisher, the editors and the reviewers. Any product that may be evaluated in this article, or claim that may be made by its manufacturer, is not guaranteed or endorsed by the publisher.
